# Association between socioeconomic status and dispensing of higher-risk drug classes and polypharmacy in older community-based populations: a nationwide cohort study

**DOI:** 10.1007/s00228-025-03896-6

**Published:** 2025-08-05

**Authors:** Juliane Frydenlund, Nicole Cosgrave, David J. Williams, Frank Moriarty, Emma Wallace, Ciara Kirke, Kathleen Bennett, Caitriona Cahir

**Affiliations:** 1 Data Science Centre, School of Population Health, RCSI University of Medicine and Health Sciences, 123 St Stephen’s Green, Dublin, D02 YN77 Ireland; 2 Department of Geriatric and Stroke Medicine, RCSI University of Medicine and Health Sciences, Dublin, Ireland; 3 School of Pharmacy and Biomolecular Sciences, RCSI University of Medicine and Health Sciences, Dublin, Ireland; 4 The Irish Longitudinal Study On Ageing (TILDA), Trinity College Dublin, Dublin, Ireland; 5 Department of General Practice, School of Medicine, University College Cork, Cork, Ireland; 6 National Quality and Patient Safety at Health Service Executive, Dublin, Ireland

**Keywords:** Socioeconomic status, Deprivation, Drug classes, Polypharmacy, Adverse drug reaction, Dispensing, Prescribing

## Abstract

**Background:**

Higher-risk medications are associated with increased risk of medication-related harm in older populations.

**Aim:**

To investigate the association between socioeconomic status (SES) and the prescribing of higher-risk drug classes and polypharmacy in older community-dwelling adults.

**Methods:**

This prospective, population-based cohort study used linked data from the Irish Longitudinal Study on Ageing (TILDA, 2018), the Health Service Executive-Primary Care Reimbursement Service (HSE-PCRS), and the General Medical Services (GMS) scheme over a 2-year follow-up. SES was measured by education, income, and private health insurance. Higher-risk drugs included antithrombotic agents, beta-blockers, calcium channel blockers, diuretics, renin–angiotensin–aldosterone system (RAAS) inhibitors, psychoanaleptics, and NSAIDs. Polypharmacy was categorised as 0–4, 5–9, and 10 + drug classes. Multivariable logistic and ordinal regression models adjusted for age, sex, and multimorbidity were used.

**Results:**

The study included 1,401 individuals aged ≥ 70 years (median age 79; 43% male); 53% had ≥ 3 chronic conditions. 43% had primary/no education, 46% had below-median income, and 55% lacked private health insurance. Antithrombotics were the most prescribed higher-risk drug (38%), and 41% had 10 + different drug classes. Higher-risk prescribing and polypharmacy were more prevalent in those with lower SES. Participants with low SES were significantly more likely to be prescribed higher-risk drugs and experience polypharmacy. The greatest association was for psychoanaleptics: adjusted OR 1.97 [95% CI: 1.32;2.95] for primary/no formal education vs. third-level education, and 1.73 [95% CI: 1.30;2.30] for no vs. private health insurance.

**Conclusion:**

SES-related disparities in higher-risk prescribing highlight the need for targeted interventions addressing social determinants of health in older populations.

**Supplementary Information:**

The online version contains supplementary material available at 10.1007/s00228-025-03896-6.

## Background

Medicines play an essential role in the treatment of illness, managing chronic conditions, maintaining health and wellbeing and improving life expectancy and quality of life. However, medication-related harm is also common, particularly in older populations and includes adverse drug reactions (ADRs) and adverse drug events (ADEs) [1]. ADRs are unintended harmful effects of a drug that occur at normal doses, ranging from mild to severe, and ADEs encompass any harm caused by medication [2, 3]. ADEs is a broader term that includes both ADRs and medication errors, such as incorrect dosages or drug interactions. Medication-related harm has been recognised globally as a critical area for improvement by the World Health Organization (WHO) who launched its third Global Patient Safety Challenge, which aims to promote and implement strategies to reduce the incidence of preventable ADRs and ADEs [4].

ADRs and ADEs can be difficult to prevent, especially in older populations, as there are many risk factors such as increasing age, frailty, multimorbidity, geriatric syndromes, cognitive impairment, and increasing levels of polypharmacy [5–8]. Specific drug classes have also been found to be associated with a higher risk of experiencing ADRs and ADEs which include antithrombotic agents, beta-blocking agents, calcium channel blockers, diuretics, renin–angiotensin–aldosterone system (RAAS), psychoanaleptics, and NSAIDs [1, 6, 9–15]. These drug classes are considered higher-risk medications in older populations and are associated with some of the most frequently reported ADRs in older populations including gastrointestinal haemorrhage, renal impairment and electrolyte disturbances [12, 14, 16, 17].

There is also evidence that socioeconomic status (SES) may influence these risk factors with people who are considered socioeconomically disadvantaged are at higher risk of cognitive impairment, multimorbidity and polypharmacy [18–21]. In a meta-analysis, participants with low SES were found to have an elevated combined risk of cognitive impairment and dementia compared to those with a higher SES [19], and low versus high education level was associated with 64% increased odds of multimorbidity [20]. Socioeconomic inequalities have also been identified in polypharmacy among older people, with people of lower SES having a significantly higher odds of polypharmacy [22].

The prevalence of potentially inappropriate prescribing has been found to be influenced by socioeconomic deprivation, with more deprived areas showing higher rates, likely due to factors such as development of complex multimorbidity earlier in life, and mental health comorbidity [23]. It is also known that individuals of high SES tend to have better access to healthcare that those of lower SES [24, 25]. However, further research is needed to explore the relationship between SES and the dispensing of higher-risk medications in older populations defined as drugs associated with medication-related harm, such as adverse drug reactions (ADRs) and adverse drug events (ADEs). While many of these medications, such as cardiovascular therapies and psychoanaleptics, may be clinically appropriate and necessary, their use still warrants careful risk–benefit assessment due to the potential for harm in older populations, particularly in the context of multimorbidity and polypharmacy.

The aim of this study was to investigate the association between SES indicators (education, income, and private health insurance) and the dispensing of higher-risk medications and higher levels of polypharmacy, in older community-based populations in Ireland.

## Methods

This study is reported using the STROBE guidelines for cohort studies [26].

### Study design and data source

This study was a cross-sectional analysis of data from a nationally representative cohort, examining associations between baseline socioeconomic status and medication dispensing over the subsequent two years. We used linked data from a national pharmacy claims database, the Health Service Executive-Primary Care Reimbursement Service (HSE-PCRS) General Medical Services (GMS) scheme, and wave 5 (2018) of The Irish LongituDinal study on Ageing (TILDA). The Irish healthcare system operates as a two-tiered public and private based system. It is predominantly tax funder but approximately forty-five percent of the population purchase private health insurance, which is assumed to secure faster access to healthcare. The HSE-PCRS GMS scheme provides free healthcare services and subsidised medications, for eligible persons (medical card holders) by means testing in Ireland; approximately 90% of the general population aged ≥ 70 years are eligible for a GMS medical card [27]. In the HSE-PCRS GMS database, pharmacy claims data are coded using the World Health Organization (WHO) Anatomical Therapeutic Chemical (ATC) classification system [28].

TILDA is a nationally representative longitudinal cohort study of community-dwelling individuals aged ≥ 50 years in Ireland. Wave-1 data collection took place between October 2009-February 2011, with follow-up assessment at two-year intervals (waves), using a computer-assisted personal interview (CAPI), self-completion questionnaire, and nurse-led health assessments to measure participants’ health, economic, and social circumstances [29–32].

Pharmacy claims data on dispensed medications was extracted for TILDA participants aged ≥ 70 years with linked HSE-PCRS GMS data from the date of interview in Wave 5 (2018) to 2 years post interview (2-year observation window).

### Socioeconomic status (SES)

The exposure of interest included three measures of participants’ SES measured per CAPI in 2018: i) educational level, ii) household income, and iii) private medical health insurance cover. Educational level was categorised according to International Standard Classification of Education as low (primary or no formal education), medium (secondary level), and high (third level) [33]. Income level was defined as above or below median annual household income after tax and deductions of the study population (25,000 euro). Private medical health insurance cover was categorised as yes or no based on individual questionnaire response.

### Higher-risk medication and polypharmacy

The outcomes of the study were higher-risk drug classes related to ADRs or ADEs (medication-related harm) [6, 10–13]. Seven different higher-risk drug classes were included: antithrombotic agents (ATC-code: B01), beta-blocking agents (ATC-code: C07), calcium channel blockers (ATC-code: C08), diuretics (ATC-code: C03), renin–angiotensin–aldosterone system (RAAS) inhibitors (ATC-code: C09), psychoanaleptics (ATC-code: N06), nonsteroidal anti-inflammatory drugs (NSAIDs) (ATC-code: M01, excluding M01AH). Each drug class plays a critical role in the management of common chronic health conditions, but their use has been linked to specific side effects, which may vary in severity (Table 1).

**Table 1:** Baseline characteristics of the study population according to educational level

	Third/higher	Secondary	Primary/none	Total	Missing
	*N* = 290	*N* = 511	*N* = 600	*N* = 1,401	*N* = 0
Male	123 (42%)	207 (41%)	276 (46%)	606 (43%)	0
Age, median (IQR)	79 (76–83)	79 (74–84)	79 (74–84)	79 (75–84)	0
Hypertension	135 (47%)	257 (50%)	309 (52%)	701 (50%)	0
Angina	21 (7%)	40 (8%)	49 (8%)	110 (8%)	0
Heart attack (including myocardial infarction or coronary thrombosis)	< 5 (< 2%)	7 (1%)	5 (1%)	16 (1%)	0
Congestive heart failure	< 5 (< 2%)	7 (1%)	12 (2%)	22 (2%)	0
Diabetes or high blood sugar	38 (13%)	62 (12%)	94 (16%)	194 (14%)	0
Stroke (cerebral vascular disease)	< 5 (< 2%)	6 (1%)	14 (2%)	24 (2%)	0
Ministroke or TIA	< 5 (< 2%)	10 (2%)	15 (2%)	28 (2%)	0
High cholesterol	103 (36%)	193 (38%)	236 (39%)	532 (38%)	0
Heart murmur	29 (10%)	36 (7%)	36 (6%)	101 (7%)	0
Atrial fibrillation	25 (9%)	38 (7%)	22 (4%)	85 (6%)	0
Abnormal heart rhythm (not atrial fibrillation)	21 (7%)	36 (7%)	39 (6%)	96 (7%)	0
Chronic lung disease such as chronic bronchitis or emphysema	13 (4%)	26 (5%)	38 (6%)	77 (5%)	0
Asthama	20 (7%)	36 (7%)	65 (11%)	121 (9%)	0
Arthritis (including osteoarthritis, or rheumatism)	142 (49%)	260 (51%)	307 (51%)	709 (51%)	0
Osteoporosis, sometimes called thin or brittle bones	72 (25%)	110 (22%)	130 (22%)	312 (22%)	0
Cancer or a malignant tumour	9 (3%)	16 (3%)	28 (5%)	53 (4%)	0
Parkinson's disease	< 5 (< 2%)	7 (1%)	< 5 (1%)	13 (1%)	0
Any emotional, nervous or psychiatric problems	12 (4%)	33 (6%)	46 (8%)	91 (6%)	0
Alzheimer's disease	< 5 (< 2%)	< 5 (< 2%)	8 (1%)	13 (1%)	0
Dementia, organic brain syndrome, senility	< 5 (< 2%)	10 (2%)	16 (3%)	27 (2%)	0
Serious memory impairment	6 (2%)	7 (1%)	13 (2%)	26 (2%)	0
Stomach ulcers	9 (3%)	11 (2%)	18 (3%)	38 (3%)	0
Varicose Ulcers (an ulcer due to varicose veins)	6 (2%)	13 (3%)	16 (3%)	35 (2%)	0
Thyroid Problems	28 (10%)	53 (10%)	64 (11%)	145 (10%)	0
Chronic kidney disease	< 5 (< 2%)	5 (1%)	< 5 (< 2%)	13 (1%)	0
Severe anaemia	5 (2%)	6 (1%)	< 5 (< 2%)	15 (1%)	0
Chest infection	21 (7%)	40 (8%)	50 (8%)	111 (8%)	0
Cataracts	44 (15%)	79 (15%)	62 (10%)	185 (13%)	0
Glaucoma	5 (2%)	10 (2%)	12 (2%)	27 (2%)	0
Age related macular degeneration	10 (3%)	9 (2%)	12 (2%)	31 (2%)	0
Other eye disease	10 (3%)	13 (3%)	20 (3%)	43 (3%)	0
Multimorbidity					
0 diseases	28 (10%)	38 (7%)	37 (6%)	103 (7%)	0
1 disease	54 (19%)	83 (16%)	101 (17%)	238 (17%)	0
2 diseases	69 (24%)	120 (23%)	134 (22%)	323 (23%)	0
3 + diseases	139 (48%)	270 (53%)	328 (55%)	737 (53%)	0

*TIA*, Transient Ischemic Attack

Polypharmacy was defined as the number of different drug classes dispensed at the 3rd level of the WHO ATC classification system over the two-year observation window. The 3rd ATC level captures therapeutic/pharmacological subgroups, allowing for a broader classification of medication use. We chose the 3rd level to ensure consistency with the classification of the higher-risk medication categories used in the main analyses, which were also defined at the 3rd ATC level.

Polypharmacy was defined as the number of drug classes at ATC level 3 during the 2-year observation window categorised into three categories: no polypharmacy (0–4 drug classes), polypharmacy (5–9 drug classes), and significant polypharmacy (10 or more drug classes). No specific drug classes were excluded, as our aim was to capture the overall medication burden rather than restrict the analysis to potentially inappropriate medications or specific therapeutic categories.

### Covariates

Covariates included age, sex, an overall measure of multimorbidity and specific medical conditions. Participants were provided with a list of 37 conditions in their CAPI and asked if a doctor had diagnosed them with any of them (Supplementary File 1). An overall measure of multimorbidity was developed as a count of these conditions; 0, 1, 2, and 3 or more long term conditions [34]. The following specific conditions were also included as covariates in models for selected higher-risk drug classes, as potential indications for the drugs; angina, atrial fibrillation, congestive heart failure, heart attack (including myocardial infarction or coronary thrombosis), high blood pressure or hypertension, ministroke or Transient Ischemic Attack (TIA), stroke (cerebral vascular disease), arthritis (including osteoarthritis, or rheumatism), Alzheimer’s disease, dementia, organic brain syndrome, senility, serious memory impairment and any emotional, nervous or psychiatric conditions.

### Statistical analyses

Baseline characteristics for the participants were presented; continues variables as median and interquartile ranges (IQR) and categorical variables as frequencies and percentages. This was presented according to educational level, income level, and having private medical health insurance. Numbers of missing observations were reported alongside these statistics. To comply with data protection regulations and ethical guidelines, cell counts fewer than 5 are suppressed and reported as'< 5'to reduce the risk of re-identification. Multivariable logistic regression was used to examine the association between educational level, household income level, and private health insurance, respectively, and each of the seven higher-risk drug classes. Ordinal regression was used for polypharmacy. Each SES indicator was separately analysed. Unadjusted and adjusted odds ratios (OR) and 95% confidence intervals are presented. The seven higher-risk drug class logistic regression models were adjusted for age, sex, specific conditions related to the drug class, and overall multimorbidity (excluding the specific conditions) (Supplementary Table 1). The polypharmacy ordinal regression model was adjusted for age, sex, and multimorbidity. Data was missing (27.8%) for household income and was imputed using multiple imputation by chained equations which included covariates and the outcome in each analysis. To assess robustness, we performed a complete case sensitivity analysis for household income. All analyses were made using performed in Stata 18 (Stata Corporation, College Station, Texas, USA).

## Results

### Characteristics of the study population by SES

In total 1,401 individuals aged ≥ 70 years were included in the study population. The median age was 79 years (IQR: 75–84), 606 (43%) were male and 737 (53%) had ≥ 3 long term conditions. The most prevalent morbidities were arthritis (*N* = 709, 51%) and hypertension (*N* = 701, 50%).

In total, 600 (43%) had primary/no formal education, 638 (46%) had an income level below the median income level and 776 (55%) had no private health insurance. The characteristics of the study population by SES are presented in Tables 1, 2, and 3. Table 1 shows that an almost similar proportion of males and females had primary/no formal education (46% males), and a lower proportion of males had secondary (41%) and third-level education (42%) compared to females. A higher proportion of females had an income below the median income level compared to males (63% vs 37%) and no health insurance (42% males) (Tables 2 and 3). Approximately half of the participants (51–57%) with primary/no formal education and/or an income below the median level and/or no private health insurance, had ≥ 3 conditions compared to 54–57% of those with higher education, income and/or health insurance (Tables 1, 2, and 3).

**Table 2:** Baseline characteristics of the study population according to income level

	Income above median	Income below median	Total	Missing
	*N* = 374	*N* = 638	*N* = 1,012	*N* = 389
Male	212 (57%)	239 (37%)	451 (45%)	155 (40%)
Age, median (IQR)	79 (75–83)	78 (74–83)	78 (74–83)	81 (76–85)
Hypertension	168 (45%)	354 (55%)	522 (52%)	179 (46%)
Angina	29 (8%)	47 (7%)	76 (8%)	34 (9%)
Heart attack (including myocardial infarction or coronary thrombosis)	< 5 (< 2%)	< 5 (< 2%)	7 (1%)	9 (2%)
Congestive heart failure	< 5 (< 2%)	10 (2%)	12 (1%)	10 (3%)
Diabetes or high blood sugar	44 (12%)	97 (15%)	141 (14%)	53 (14%)
Stroke (cerebral vascular disease)	5 (1%)	7 (1%)	12 (1%)	12 (3%)
Ministroke or TIA	6 (2%)	11 (2%)	17 (2%)	11 (3%)
High cholesterol	154 (41%)	246 (39%)	400 (40%)	132 (34%)
Heart murmur	33 (9%)	43 (7%)	76 (8%)	25 (6%)
Atrial fibrillation	26 (7%)	32 (5%)	58 (6%)	27 (7%)
Abnormal heart rhythm (not atrial fibrillation)	28 (7%)	40 (6%)	68 (7%)	28 (7%)
Chronic lung disease such as chronic bronchitis or emphysema	14 (4%)	33 (5%)	47 (5%)	30 (8%)
Asthama	32 (9%)	63 (10%)	95 (9%)	26 (7%)
Arthritis (including osteoarthritis, or rheumatism)	179 (48%)	350 (55%)	529 (52%)	180 (46%)
Osteoporosis, sometimes called thin or brittle bones	69 (18%)	156 (24%)	225 (22%)	87 (22%)
Cancer or a malignant tumour	12 (3%)	28 (4%)	40 (4%)	13 (3%)
Parkinson's disease	< 5 (< 2%)	< 5 (< 2%)	8 (1%)	5 (1%)
Any emotional, nervous or psychiatric problems	15 (4%)	50 (8%)	65 (6%)	26 (7%)
Alzheimer's disease	< 5 (< 2%)	< 5 (< 2%)	< 5 (0%)	10 (3%)
Dementia, organic brain syndrome, senility	< 5 (< 2%)	5 (1%)	9 (1%)	18 (5%)
Serious memory impairment	< 5 (< 2%)	5 (1%)	8 (1%)	18 (5%)
Stomach ulcers	7 (2%)	19 (3%)	26 (3%)	12 (3%)
Varicose Ulcers (an ulcer due to varicose veins)	5 (1%)	24 (4%)	29 (3%)	6 (2%)
Thyroid Problems	37 (10%)	68 (11%)	105 (10%)	40 (10%)
Chronic kidney disease	< 5 (< 2%)	< 5 (< 2%)	5 (0%)	8 (2%)
Severe anaemia	< 5 (< 2%)	6 (1%)	8 (1%)	7 (2%)
Chest infection	35 (9%)	57 (9%)	92 (9%)	19 (5%)
Cataracts	52 (14%)	82 (13%)	134 (13%)	51 (13%)
Glaucoma	6 (2%)	10 (2%)	16 (2%)	11 (3%)
Age related macular degeneration	9 (2%)	12 (2%)	21 (2%)	10 (3%)
Other eye disease	11 (3%)	25 (4%)	36 (4%)	7 (2%)
Multimorbidity				
0 diseases	35 (9%)	40 (6%)	75 (7%)	28 (7%)
1 disease	68 (18%)	86 (13%)	154 (15%)	84 (22%)
2 diseases	90 (24%)	150 (24%)	240 (24%)	83 (21%)
3 + diseases	181 (48%)	362 (57%)	543 (54%)	194 (50%)

*TIA*, Transient Ischemic Attack

**Table 3:** Baseline characteristics of the study population according to private health insurance

	Yes private medical insurance	No private medical insurance	Total	Missing
	*N* = 619	*N* = 776	*N* = 1,395	*N* = 6
Male	280 (45%)	325 (42%)	605 (43%)	1 (17%)
Age, median (IQR)	80 (76–83)	78 (74–84)	79 (75–84)	86 (83–86)
Hypertension	284 (46%)	415 (53%)	699 (50%)	2 (33%)
Angina	41 (7%)	68 (9%)	109 (8%)	1 (17%)
Heart attack (including myocardial infarction or coronary thrombosis)	4 (1%)	12 (2%)	16 (1%)	0 (0%)
Congestive heart failure	< 5 (< 2%)	19 (2%)	22 (2%)	0 (0%)
Diabetes or high blood sugar	66 (11%)	127 (16%)	193 (14%)	1 (17%)
Stroke (cerebral vascular disease)	5 (1%)	18 (2%)	23 (2%)	1 (17%)
Ministroke or TIA	9 (1%)	19 (2%)	28 (2%)	0 (0%)
High cholesterol	244 (39%)	287 (37%)	531 (38%)	1 (17%)
Heart murmur	50 (8%)	51 (7%)	101 (7%)	0 (0%)
Atrial fibrillation	45 (7%)	39 (5%)	84 (6%)	1 (17%)
Abnormal heart rhythm (not atrial fibrillation)	42 (7%)	54 (7%)	96 (7%)	0 (0%)
Chronic lung disease such as chronic bronchitis or emphysema	24 (4%)	52 (7%)	76 (5%)	1 (17%)
Asthama	53 (9%)	68 (9%)	121 (9%)	0 (0%)
Arthritis (including osteoarthritis, or rheumatism)	307 (50%)	402 (52%)	709 (51%)	0 (0%)
Osteoporosis, sometimes called thin or brittle bones	148 (24%)	164 (21%)	312 (22%)	0 (0%)
Cancer or a malignant tumour	23 (4%)	30 (4%)	53 (4%)	0 (0%)
Parkinson's disease	6 (1%)	7 (1%)	13 (1%)	0 (0%)
Any emotional, nervous or psychiatric problems	30 (5%)	61 (8%)	91 (7%)	0 (0%)
Alzheimer's disease	< 5 (< 2%)	12 (2%)	13 (1%)	0 (0%)
Dementia, organic brain syndrome, senility	7 (1%)	19 (2%)	26 (2%)	1 (17%)
Serious memory impairment	5 (1%)	20 (3%)	25 (2%)	1 (17%)
Stomach ulcers	12 (2%)	26 (3%)	38 (3%)	0 (0%)
Varicose Ulcers (an ulcer due to varicose veins)	13 (2%)	21 (3%)	34 (2%)	1 (17%)
Thyroid Problems	66 (11%)	78 (10%)	144 (10%)	1 (17%)
Chronic kidney disease	6 (1%)	7 (1%)	13 (1%)	0 (0%)
Severe anaemia	6 (1%)	9 (1%)	15 (1%)	0 (0%)
Chest infection	48 (8%)	61 (8%)	109 (8%)	2 (33%)
Cataracts	92 (15%)	92 (12%)	184 (13%)	1 (17%)
Glaucoma	9 (1%)	17 (2%)	26 (2%)	1 (17%)
Age related macular degeneration	19 (3%)	11 (1%)	30 (2%)	1 (17%)
Other eye disease	19 (3%)	23 (3%)	42 (3%)	1 (17%)
Multimorbidity				
0 diseases	54 (9%)	49 (6%)	103 (7%)	0 (0%)
1 disease	102 (16%)	134 (17%)	236 (17%)	2 (33%)
2 diseases	146 (24%)	176 (23%)	322 (23%)	1 (17%)
3 + diseases	317 (51%)	417 (54%)	734 (53%)	3 (50%)

*TIA*, Transient Ischemic Attack

Among individuals of lower SES (primary/no formal education, below median income, no private health insurance) 46–55% had hypertension compared to 41–46% in those with a higher SES (secondary/third-level education, above median income and health insurance). 47–55% had arthritis compared to 48–55% in those with a higher SES (Tables 1, 2, and 3).

### Higher-risk medications and polypharmacy by SES

Table 4 shows that antithrombotic agents were the most frequently prescribed higher-risk drug class (*N* = 532, 38%), and that 577 (41%) participants had significant polypharmacy. Across all the seven higher-risk drug classes, there was a higher proportion of participants with primary/no formal education (45–50%) compared to those with secondary level (36–39%) and third-level education (13–17%). There were similar findings for income and private health insurance with 65–70% of participants with below median income prescribed higher-risk drug classes compared to 30–35% of participants with above median income and 58–67% of participants with no private health insurance prescribed higher-risk drug classes compared to 35–42% of those with private health insurance. The prevalence of significant polypharmacy (≥ 10 drugs) was higher in those with primary/no formal education (47%), below median income (68%) and no health insurance (61%) compared to those with higher education (secondary 36%, third-level 16%), above median income (32%) and health insurance (39%).

**Table 4:** Characteristics of the higher-risk drug classes and polypharmacy by SES

	No antithrombotic agents	Antithrombotic agents	No beta-blocking agents	Beta-blocking agents	No blockers	Blockers	No diuretics	Diuretics	No RAAS	RAAS	No psychoanaleptics	Psychoanaleptics	No NSAID	NSAID	Non-polypharmacy	Polypharmacy	Sig. polypharmacy
	*N* = 869	*N* = 532	*N* = 1,079	*N* = 322	*N* = 1,129	*N* = 272	*N* = 1,135	*N* = 266	*N* = 935	*N* = 466	*N* = 1,131	*N* = 270	*N* = 1,081	*N* = 320	*N* = 540	*N* = 284	*N* = 577
Educational level																	
Third/higher	206 (24%)	84 (16%)	236 (22%)	54 (17%)	248 (22%)	42 (15%)	245 (22%)	45 (17%)	218 (23%)	72 (15%)	254 (22%)	36 (13%)	238 (22%)	52 (16%)	146 (27%)	50 (18%)	94 (16%)
Secondary	310 (36%)	201 (38%)	387 (36%)	124 (39%)	410 (36%)	101 (37%)	416 (37%)	95 (36%)	339 (36%)	172 (37%)	413 (37%)	98 (36%)	399 (37%)	112 (35%)	187 (35%)	114 (40%)	210 (36%)
Primary/none	353 (41%)	247 (46%)	456 (42%)	144 (45%)	471 (42%)	129 (47%)	474 (42%)	126 (47%)	378 (40%)	222 (48%)	464 (41%)	136 (50%)	444 (41%)	156 (49%)	207 (38%)	120 (42%)	273 (47%)
Income level																	
Above median income	229 (39%)	145 (35%)	288 (38%)	86 (33%)	299 (37%)	75 (35%)	306 (38%)	68 (33%)	259 (40%)	115 (32%)	312 (39%)	62 (30%)	290 (38%)	84 (33%)	149 (44%)	78 (35%)	147 (32%)
Below median income	364 (61%)	274 (65%)	463 (62%)	175 (67%)	500 (63%)	138 (65%)	501 (62%)	137 (67%)	390 (60%)	248 (68%)	495 (61%)	143 (70%)	469 (62%)	169 (67%)	187 (56%)	145 (65%)	306 (68%)
Health insurance																	
Yes private medical insurance	398 (46%)	221 (42%)	490 (46%)	129 (40%)	517 (46%)	102 (38%)	526 (47%)	93 (35%)	446 (48%)	173 (37%)	531 (47%)	88 (33%)	486 (45%)	133 (42%)	277 (52%)	115 (40%)	227 (39%)
No private medical insurance	465 (54%)	311 (58%)	583 (54%)	193 (60%)	607 (54%)	169 (62%)	603 (53%)	173 (65%)	484 (52%)	292 (63%)	595 (53%)	181 (67%)	590 (55%)	186 (58%)	258 (48%)	169 (60%)	349 (61%)

### Association between SES and higher-risk medications and polypharmacy

**Fig. 1:**
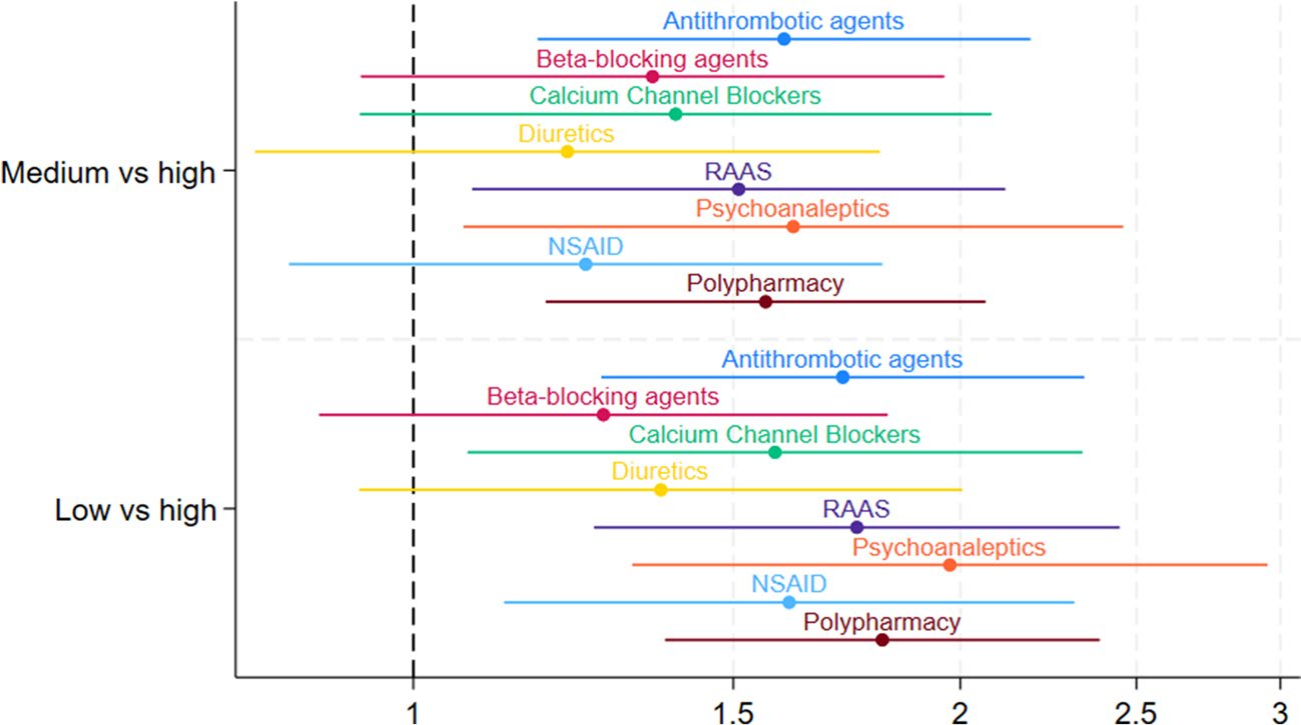
Forest plot of OR and 95% CI of the multivariate logistic regressions of educational level associated with the dispensing of drug classes (logarithmic scale). Adjustments: Antithrombotic agents/B01: gender, age, heart attack, atrial fibrillation, stroke, TIA, multimorbidity; Beta-blocking agents/C07: gender, age, angina, congestive heart failure, high blood pressure or hypertension, multimorbidity; Calcium channel blockers/C08: gender, age, angina, high blood pressure or hypertension, multimorbidity; Diuretics/C03: gender, age, congestive heart failure, high blood pressure or hypertension, multimorbidity; RAAS/C09: gender, age, congestive heart failure, high blood pressure or hypertension, multimorbidity; Psychoanaleptics/N06: gender, age, nervous or psychiatric conditions, ‘Alzheimer's disease or dementia, organic brain syndrome, senility’, serious memory impairment, multimorbidity; NSAID: gender, age, arthritis, multimorbidity; Polypharmacy: gender, age, multimorbidity

Figure 1 shows that participants with primary or no formal education were significantly more likely to be prescribed antithrombotic agents, calcium channel blockers, RAAS, psychoanaleptics, and NSAIDs compared to those with third-level education ranging from adj. OR 1.58 [95% CI: 1.07;2.33] to adj. OR 1.97 [95%CI: 1.32;2.95]. Those with secondary educational level were significantly more likely to be prescribed antithrombotic agents, RAAS, and psychoanaleptics compared to those with third-level education ranging from adj. OR 1.51 [95%CI: 1.08;2.12] and adj. OR 1.62 [95%CI: 1.07;2.46].

**Fig. 2:**
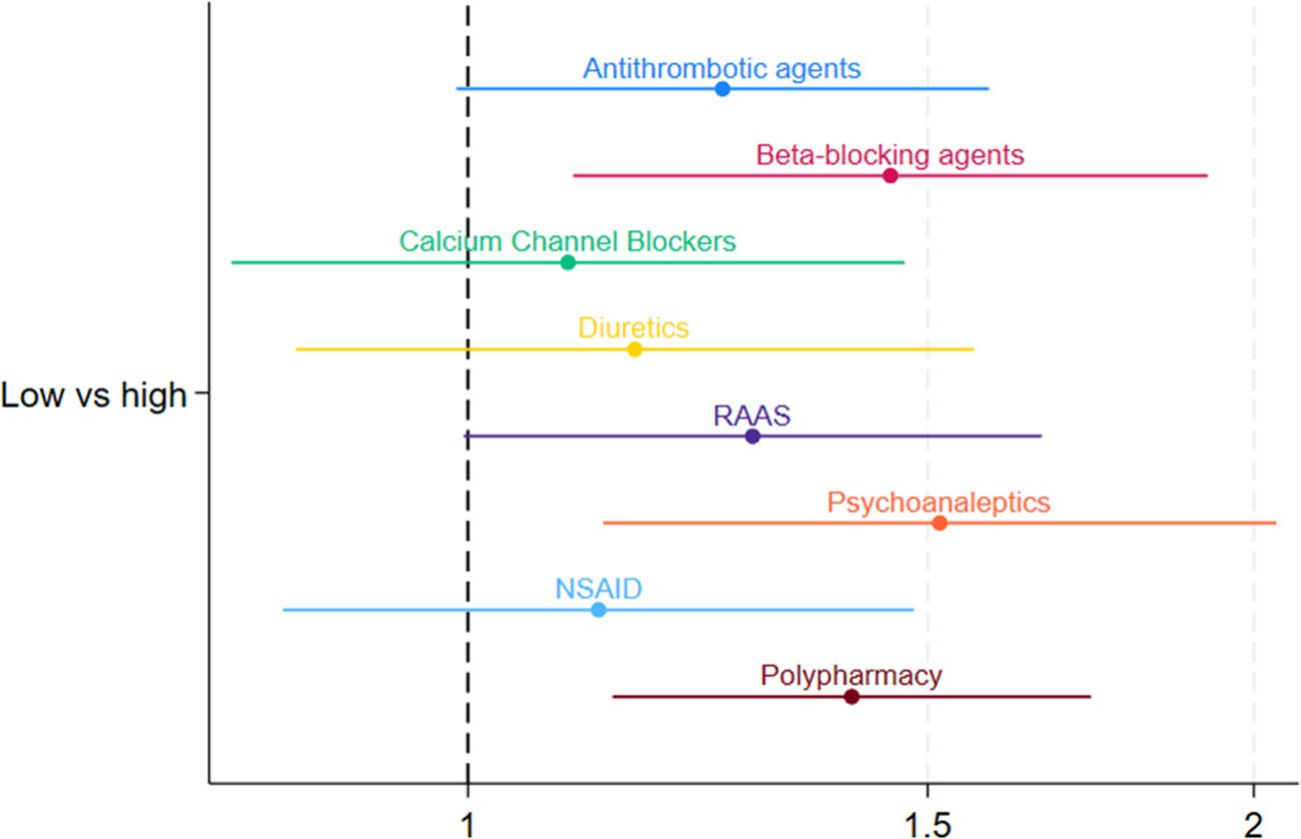
Forest plot of OR and 95% CI of the multivariate logistic regressions of income level associated with the dispensing of drug classes (logarithmic scale). Adjustments: Antithrombotic agents/B01: gender, age, heart attack, atrial fibrillation, stroke, TIA, multimorbidity; Beta-blocking agents/C07: gender, age, angina, congestive heart failure, high blood pressure or hypertension, multimorbidity; Calcium channel blockers/C08: gender, age, angina, high blood pressure or hypertension, multimorbidity; Diuretics/C03: gender, age, congestive heart failure, high blood pressure or hypertension, multimorbidity; RAAS/C09: gender, age, congestive heart failure, high blood pressure or hypertension, multimorbidity; Psychoanaleptics/N06: gender, age, nervous or psychiatric conditions, ‘Alzheimer's disease or dementia, organic brain syndrome, senility’, serious memory impairment, multimorbidity; NSAID: gender, age, arthritis, multimorbidity; Polypharmacy: gender, age, multimorbidity

**Fig. 3:**
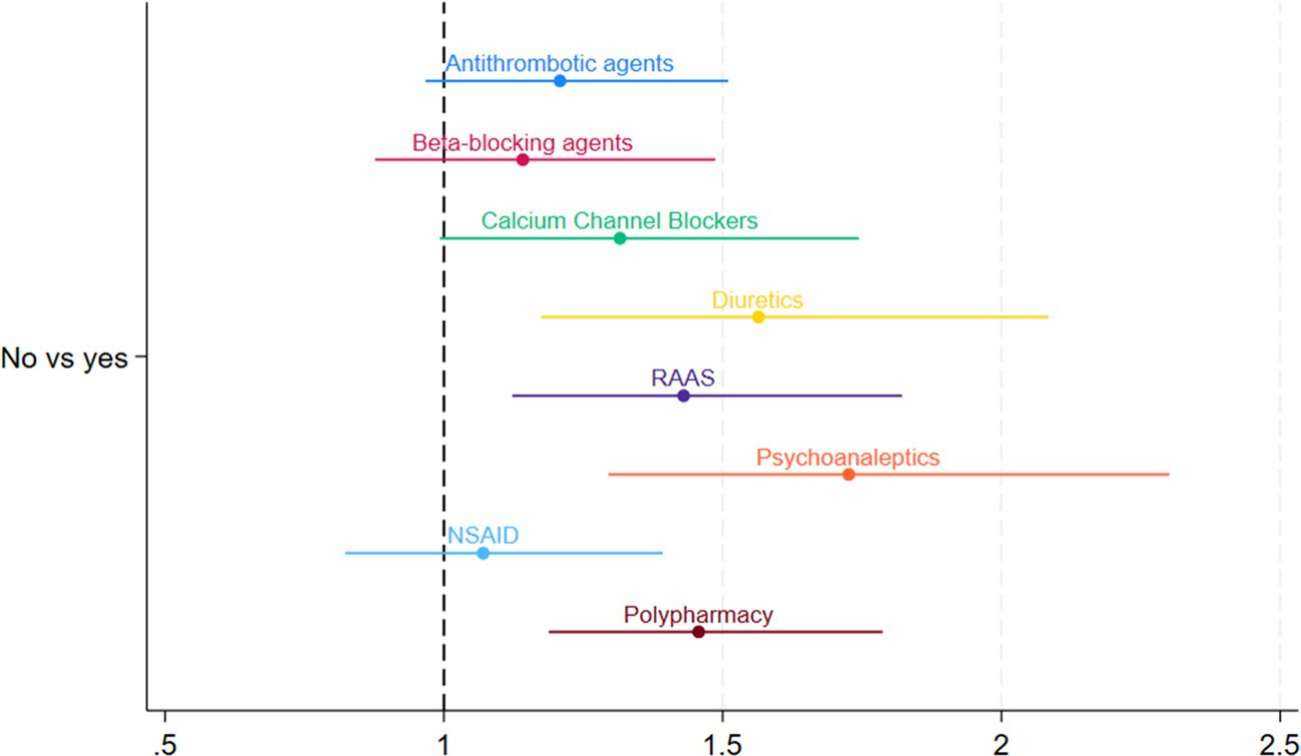
Forest plot of OR and 95% CI of the multivariate logistic regressions of private health insurance level associated with the dispensing of drug classes (logarithmic scale). Adjustments: Antithrombotic agents/B01: gender, age, heart attack, atrial fibrillation, stroke, TIA, multimorbidity; Beta-blocking agents/C07: gender, age, angina, congestive heart failure, high blood pressure or hypertension, multimorbidity; Calcium channel blockers/C08: gender, age, angina, high blood pressure or hypertension, multimorbidity; Diuretics/C03: gender, age, congestive heart failure, high blood pressure or hypertension, multimorbidity; RAAS/C09: gender, age, congestive heart failure, high blood pressure or hypertension, multimorbidity; Psychoanaleptics/N06: gender, age, nervous or psychiatric conditions, ‘Alzheimer's disease or dementia, organic brain syndrome, senility’, serious memory impairment, multimorbidity; NSAID: gender, age, arthritis, multimorbidity; Polypharmacy: gender, age, multimorbidity

Figure 2 shows that income below the median was not significantly associated with any of the higher risk drug classes. Figure 3 showed that those having no private health insurance were significantly more likely to be prescribed diuretics, RAAS, and psychoanaleptics ranging from adj. OR 1.43 [95%CI: 1.12;1.82] to adj. OR 1.73 [95%CI: 1.30;2.30].

For polypharmacy, participants with primary/no education were more likely (adj. OR 1.81 [95%CI: 1.38;2.39]), to experience polypharmacy compared to those with third-level education. Participants with household income below the median, also were more likely to experience polypharmacy (adj. OR 1.39 [95%CI: 1.07;1.80]) compared to those with income above the median. Participants without private health insurance were also more likely to experience polypharmacy (adj. OR 1.46 [95%CI: 1.19;1.79]) compared to those with private health insurance.

Unadjusted and adjusted results of all analyses are presented in Supplementary Table 2.

The complete case sensitivity analysis for household income showed that polypharmacy results remained consistent and statistically significant, while associations for individual drug classes became non-significant, likely due to reduced statistical power and potential selection bias from excluding cases with missing income data (Supplementary Table 3).

## Discussion

This study found that older community-based populations with lower SES (including lower educational level, household income below the median and not having private health insurance) were more likely to be prescribed higher-risk drug classes and to experience significant polypharmacy. This may indicate that older people with a lower SES may have a heightened risk of experiencing medication-related harm, including ADRs and ADEs.

Few studies have investigated the association between SES and specific higher-risk drug classes known to be related with higher risk of ADRs and ADEs and the findings to date are mixed. A study based in the UK found an association between socioeconomic deprivation and overprescribing of higher-risk medication in general practice which varied by region and drug type, and found that geographical location is associated with overprescribing, independent of SES [35]. A study investigating the prescribing patterns of antithrombotic agents and beta-blockers, in deprived areas in Hungary found that prescription and redemption rates were higher in more deprived areas [36]. While an Australian and New Zealand study investigating the influence of prescriber factors on antithrombotic prescribing found that SES had no statistical impact on clinician prescribing choices in a discrete choice experiment [37].

A study of trends and inequities in beta-blocker prescribing for heart failure found that women were less likely to receive treatment, compared to men, and patients residing in areas of deprivation were also less likely to receive treatment, compared to those in the most affluent areas [38]. Regarding the prescribing of NSAIDs, a Danish study of 407,330 individuals found that low SES was associated with greater initiation and repeat prescriptions of NSAIDs, in relation to cardiovascular events, and concluded that lifestyle and socioeconomic position should be considered as potential confounders in studies examining the impact of NSAID use on cardiovascular events or mortality [39]. Another Danish study of 103,209 patients undergoing total hip arthroplasty found that markers of low SES were associated with higher use of analgesics (NSAIDs and opioids) both before and after total hip arthroplasty and there was lower reduction in use of analgesics after total hip arthroplasty compared with markers of high SES [40]. Studies in Spain and Sweden have found higher rates of use of antipsychotics and the greater likelihood of receiving this type of drug, as well less specialised psychiatric prescribing of these drugs, among people with low SES [41–43].

Previous research has indicated that lower SES is associated with the increased prescribing of medication in general as patients with public health coverage tend to be prescribed more medications [44–46]. Older adults from lower socioeconomic backgrounds have also been reported to have a higher risk of polypharmacy and potentially inappropriate prescribing [22]. Our results showed that lower SES was associated with both a higher likelihood of significant polypharmacy and the prescribing of higher-risk drug classes associated with medication-related harm [47].

### Limitations

This study used data from a large national population‐based pharmacy claims database (HSE-PCRS) linked to a nationally representative, longitudinal cohort study, for those aged ≥ 70 years in Ireland (TILDA). However, a small proportion of this cohort were not eligible for a medical card due to having a high-income level and were excluded from the study; the cohort may not be representative of the very wealthy older population [48]. Also, a considerable proportion of data was also missing for income level which was managed using multiple imputation.

The sensitivity analysis showed that the association between lower SES and polypharmacy was robust to the handling of missing data. However, the associations with specific higher-risk drug classes became non-significant in the complete case analysis. This may reflect reduced power due to sample size reduction or bias introduced by non-random missingness of income data. Therefore, findings for individual drug classes should be interpreted with caution.

As a robustness check, we also calculated polypharmacy using the 5th ATC level (chemical substance/active ingredient), which is commonly used in polypharmacy research. This alternative definition produced similar results, with no substantial change in participant classification or in the observed associations. Therefore, we retained the 3rd level measure for conceptual consistency across the study. We also chose to include all systemic drug classes in the polypharmacy measure without exclusions, reflecting the real-world medication burden experienced by older adults. This approach is consistent with our focus on overall medication exposure rather than on inappropriate prescribing alone.

Our results showed that individuals of lower SES were more likely to be prescribed higher-risk drug classes and to experience significant polypharmacy which could be explained by the fact that these individuals had higher levels of morbidity. We adjusted for a wide range of chronic conditions and multimorbidity to account for differences in disease burden, acknowledging that these conditions are directly related to medication need. However, the medical condition data were based on self-reported questionnaires rather than primary care or hospital records, which introduces the risk of misclassification. This limitation may be particularly relevant for individuals with cognitive impairment or lower educational attainment, who may have had more difficulty accurately reporting their health conditions. Such non-random underreporting of comorbidities could lead to residual confounding, potentially exaggerating the association between SES and medication use. Furthermore, we were unable to determine disease severity or progression, which could also influence prescribing patterns. For example, individuals with lower SES may have had more advanced stages of illness, requiring multiple medications within the same therapeutic class. While our aim was to adjust for differences in morbidity to reduce confounding by indication, we acknowledge that these limitations may have impacted the findings and should be considered when interpreting the results.

The fact that some patients are at risk of being prescribed higher-risk drug classes does not directly indicate overprescribing or inappropriate prescribing, as we did not investigate the indication for the use of the various different drug-classes.

Neither did we investigate interactions between the drug classes or the relationship between them. Future research should investigate these relationships in order to provide a more nuanced picture of the effect of SES on drug utilisation but also to ensure that individuals of low SES are not at a higher risk of potential adverse medication-related harm health outcomes compared to those of higher SES.

### Implications

It has been found that individuals residing in socioeconomically disadvantaged areas experience the onset of multimorbidity at an earlier age and accumulating comorbid conditions at a more rapid pace which will impact on condition severity as the person ages, and this then impacts on prescriptions [49, 50]. This phenomenon is further compounded by the higher prevalence of key risk factors for chronic diseases, such as smoking and obesity [51]. Additionally, the severity of multimorbidity is particularly pronounced among individuals with severe mental illness, which makes management of physical conditions more challenging [52]. For certain drug classes, such as antithrombotics, antihypertensives (including RAAS inhibitors), and antidepressants, prescribing and adherence are generally beneficial, as their use has significant impacts on reducing morbidity and mortality [53–55]. However, these medications are also associated with ADRs, necessitating a careful balance of risk and benefit [13]. Regular monitoring, medication reviews, and adjustments are required, particularly when new drugs are introduced [56]. In the case of NSAIDs, while there remains a benefit-risk consideration, the risk is heightened in older populations, making the need for careful evaluation even more critical [57]. Regarding psycholeptics, specifically antipsychotics, their use is necessary for treating psychiatric conditions; however, their benefits may be outweighed by risks when used for other indicators such as behavioural symptoms in older adults with conditions such as dementia [58]. Similarly, in prescribing sedatives and anxiolytics, careful consideration of the balance of clinical risks and benefits, as well as patient preferences, is required in older patients [59].

Regular structured medication review is an intervention that may reduce the risk of ADRs and other medication-related harm, however, for patients with poor health literacy these consultations can be more difficult to undertake [60–62]. People living in areas of socio-economic disadvantage are more exposed to health risks due to an increased risk of poorer work conditions, poor housing, and limited access to healthcare, healthy food, and recreational activities [63]. Access to quality health care is also more challenging, which can delay diagnosis and treatment and therefore worsen outcomes. Early life disadvantages, like poor nutrition or education, can also lead to poorer health later on, creating a cycle of growing health disparities across generations [64]. This can be further compounded by healthcare challenges including prescriber conservatism, treatment guidelines focused on single diseases rather than multimorbidity, and an imbalance between specialists and generalists [65]. Furthermore, inadequate access to psychological treatments and barriers to deprescribing psychiatric medications can further complicate care, and limited health literacy and low numeracy impact patients'ability to engage in shared decision-making, self-manage medications, and adhere to treatment plans [65].

Our findings show that SES is associated with the dispensing of higher-risk medications and polypharmacy, even after adjusting for clinical factors. However, we agree that clinical need is the primary determinant of prescribing, and interventions such as medication reviews should prioritise patients based on clinical risk. However, the persistence of SES associations suggests that social factors contribute to disparities in medication exposure beyond disease burden alone. From a public health perspective, this highlights the need for equity-focused strategies to ensure that medication management is safe and appropriate across all socioeconomic groups.

Awareness of social determinants of health such as SES may allow clinicians to create more personalised and effective treatment plans. The International Group for Reducing Inappropriate Medication Use & Polypharmacy has suggested a number of interventions that can reduce over-prescribing of medication and recommends identifying subgroups that would most benefit from these interventions [66]. Older people from lower SES groups should be prioritised as a subgroup for mediation reviews and other interventions to manage the use of higher-risk drugs and potential over-prescribing. Addressing the use of higher-risk drug classes and polypharmacy in those from lower SES requires a comprehensive approach that considers not only the medications prescribed but also the patient’s overall health status, comorbidities, and social context and healthcare resources should be allocated accordingly.

## Conclusion

This study found that individuals with low SES, particularly those with lower formal education, lower income, and without private health insurance, were more likely to be prescribed higher-risk drug classes and to experience significant polypharmacy. These findings point to the need for targeted efforts to reduce medication-related health disparities among people with lower SES. This includes improving access to healthcare, providing regular medication reviews, and supporting health literacy. Because individuals with low SES are also more likely to have multiple and more severe health conditions, addressing broader factors like smoking, obesity, and social conditions, e.g. housing etc., is essential for improving health outcomes.

## Supplementary Information

Below is the link to the electronic supplementary material.

Supplementary file1 (DOCX 17 KB)

## Data Availability

Data is available on completion of a request form (see https://tilda.tcd.ie/data/accessing-data/). The data are not publicly available in their raw form due to them containing information that could compromise research participant privacy/consent.

## Abbreviations

ADE: Adverse drug event
ADR: Adverse drug reactions
ATC: Anatomical Therapeutic Chemical
CAPI: Computer-assisted personal interview
CI: Confidence interval
GMS: General Medical Services
HSE-PCRS: Health Service Executive-Primary Care Reimbursement Service
NSAID: Non-steroidal anti-inflammatory drugs
OR: Odds ratio
RAAS: Renin–angiotensin–aldosterone system
SES: Socioeconomic status
TIA: Transient Ischemic Attack
TILDA: The Irish Longitudinal Study on Ageing
WHO: World Health Organization
