# Supplementary material for: Association between socioeconomic status and dispensing of higher-risk drug classes and polypharmacy in older community-based populations: a nationwide cohort study
Source: Eur J Clin Pharmacol. 2025 Aug 5;81(11):1609–22. doi: 10.1007/s00228-025-03896-6 (PMC12511149; doi:10.1007/s00228-025-03896-6)
Supplement: Supplementary file 1 — Supplementary file1 (DOCX 17 KB) [file 228_2025_3896_MOESM1_ESM.docx]

**Supplementary Table 3:** Sensitivity analysis comparing complete case results for household income with the main multiple imputation analysis. The direction and magnitude of associations were similar, supporting the robustness of the primary findings.

|  | **Antithrombotic agents B01** | | **Beta-blocking agents C07** | | **Calcium Channel Blockers C08** | | **Diuretics C03** | |
| --- | --- | --- | --- | --- | --- | --- | --- | --- |
|  | OR [95%CI] | Adj. OR [95%CI] | OR [95%CI] | Adj. OR [95%CI] | OR [95%CI] | Adj. OR [95%CI] | OR [95%CI] | Adj. OR [95%CI] |
|  | N=1,012 | N=1,012 | N=1,012 | N=1,012 | N=1,012 | N=1,012 | N=1,012 | N=1,012 |
| **Income level** |  |  |  |  |  |  |  |  |
| Above  median | Ref. | Ref. | Ref. | Ref. | Ref. | Ref. | Ref. | Ref. |
| Below median | 1.19 [0.92;1.54] | 1.30 [0.99;1.71] | 1.27 [0.94;1.70] | 1.29 [0.94;1.76] | 1.10 [[0.80;1.51] | 1.00 [0.71;1.39] | 1.23 [0.89;1.70] | 1.14 [0.81;1.61] |

Adjustments: B01: gender, age, heart attack, atrial fibrillation, stroke, TIA, multimorbidity; C07: gender, age, angina, congestive heart failure, high blood pressure or hypertension, multimorbidity; C08: gender, age, angina, high blood pressure or hypertension, multimorbidity; C03: gender, age, congestive heart failure, high blood pressure or hypertension, multimorbidity; C09: gender, age, congestive heart failure, high blood pressure or hypertension, multimorbidity; N06: gender, age, nervous or psychiatric conditions, ‘Alzheimer's disease or dementia, organic brain syndrome, senility’, serious memory impairment, multimorbidity; NSAID: gender, age, arthritis, multimorbidity; polypharmacy: gender, age, multimorbidity

Supplementary Table 3 continued

|  | **RAAS C09** | | **Psychoanaleptics N06** | | **NSAID** | | **Polypharmacy** | |
| --- | --- | --- | --- | --- | --- | --- | --- | --- |
|  | OR [95%CI] | Adj. OR [95%CI] | OR [95%CI] | Adj. OR [95%CI] | OR [95%CI] | Adj. OR [95%CI] | OR [95%CI] | Adj. OR [95%CI] |
|  | N=1,012 | N=1,012 | N=1,012 | N=1,012 | N=1,012 | N=1,012 | N=1,012 | N=1,012 |
| **Income level** |  |  |  |  |  |  |  |  |
| Above  median | Ref. | Ref. | Ref. | Ref. | Ref. | Ref. | Ref. | Ref. |
| Below median | 1.43 [1.09;1.88] | 1.30 [0.97;1.75] | 1.45 [1.04;2.02] | 1.30 [0.92;1.82] | 1.24 [0.92;1.68] | 1.11 [0.81;1.51] | 1.50 [1.18;1.91] | 1.41 [1.10;1.81] |

Adjustments: B01: gender, age, heart attack, atrial fibrillation, stroke, TIA, multimorbidity; C07: gender, age, angina, congestive heart failure, high blood pressure or hypertension, multimorbidity; C08: gender, age, angina, high blood pressure or hypertension, multimorbidity; C03: gender, age, congestive heart failure, high blood pressure or hypertension, multimorbidity; C09: gender, age, congestive heart failure, high blood pressure or hypertension, multimorbidity; N06: gender, age, nervous or psychiatric conditions, ‘Alzheimer's disease or dementia, organic brain syndrome, senility’, serious memory impairment, multimorbidity; NSAID: gender, age, arthritis, multimorbidity; polypharmacy: gender, age, multimorbidity
